# Decitabine Treatment of Glioma-Initiating Cells Enhances Immune Recognition and Killing

**DOI:** 10.1371/journal.pone.0162105

**Published:** 2016-08-31

**Authors:** Cristina Riccadonna, Céline Yacoub Maroun, Romain Vuillefroy de Silly, Margaux Boehler, Marta Calvo Tardón, Simone Jueliger, Pietro Taverna, Leticia Barba, Eliana Marinari, Serena Pellegatta, Esen Yonca Bassoy, Denis Martinvalet, Pierre-Yves Dietrich, Paul R. Walker

**Affiliations:** 1 Centre of Oncology, Geneva University Hospitals and University of Geneva, Geneva, Switzerland; 2 Astex Pharmaceuticals, Cambridge Science Park, Cambridge, United Kingdom; 3 Astex Pharmaceuticals, Pleasanton, CA, United States of America; 4 Unit of Molecular Neuro-Oncology, Fondazione I.R.C.C.S. Istituto Neurologico C. Besta, Milan, Italy; 5 Department of Cell Physiology and Metabolism, University of Geneva, Geneva, Switzerland; Cedars-Sinai Medical Center, UNITED STATES

## Abstract

Malignant gliomas are aggressive brain tumours with very poor prognosis. The majority of glioma cells are differentiated (glioma-differentiated cells: GDCs), whereas the smaller population (glioma-initiating cells, GICs) is undifferentiated and resistant to conventional therapies. Therefore, to better target this pool of heterogeneous cells, a combination of diverse therapeutic approaches is envisaged. Here we investigated whether the immunosensitising properties of the hypomethylating agent decitabine can be extended to GICs. Using the murine GL261 cell line, we demonstrate that decitabine augments the expression of the death receptor FAS both on GDCs and GICs. Interestingly, it had a higher impact on GICs and correlated with an enhanced sensitivity to FASL-mediated cell death. Moreover, the expression of other critical molecules involved in cognate recognition by cytotoxic T lymphocytes, MHCI and ICAM-1, was upregulated by decitabine treatment. Consequently, T-cell mediated killing of both GDCs and GICs was enhanced, as was T cell proliferation after reactivation. Overall, although GICs are described to resist classical therapies, our study shows that hypomethylating agents have the potential to enhance glioma cell recognition and subsequent destruction by immune cells, regardless of their differentiation status. These results support the development of combinatorial treatment modalities including epigenetic modulation together with immunotherapy in order to treat heterogenous malignancies such as glioblastoma.

## Introduction

Malignant gliomas are highly invasive and heterogeneous brain tumours. Among the different types of gliomas, glioblastomas (GBMs) represent the most deadly primary brain tumours [1]. Despite conventional treatment, i.e. radiotherapy and concomitant Temozolomide (TMZ) chemotherapy, GBM patient survival remains dismal [2]. One hallmark of glioma is its high heterogeneity [3, 4]; consequently, the use of new combined treatments is envisaged in order to target cells that are likely to respond differently to therapy.

The majority of glioma cells are differentiated (glioma differentiated cells, GDCs), whereas a small fraction (glioma-initiating cells, GICs) is undifferentiated and likely to be the cause of tumour resistance to conventional therapies [5–8]. Human GICs were characterised by their stem-like marker expression and their potential to initiate aggressive tumours when orthotopically xenografted in immunodeficient mice [7]. Overall, GICs are predicted to play an important role in glioma initiation and recurrence, and represent a relevant target for future therapeutic approaches.

Malignant gliomas are frequently infiltrated by immune cells [9], but this spontaneous immune response is insufficient to control tumour growth. Various immunotherapy strategies have therefore been explored, with T cell adoptive immunotherapy being of particular interest [10]. Furthermore, diverse new glioma-associated antigens have been characterised that could allow the use of cytotoxic CD8 T cells (CTLs) to preferentially target glioma cells, limiting off-target side effects. However, T-cell based immunotherapy is unlikely to be optimal unless glioma cells are made more sensitive to immune attacks by increasing their visibility or sensitivity to immune effector mechanisms, or by reducing immunosuppressive factors [11, 12].

Anti-neoplastic agents are now understood to not only act directly on the tumour, but also to potentiate anti-tumoural immune responses and/or to modulate immune escape mechanisms. Many studies reported that the efficacy of some anti-neoplastic agents relied on the particular host immune compartment impacted—e.g. tumour-infiltrating MDSCs and Tregs [13, 14]. Alternatively, anti-neoplastic agents may also favour immune reactions by increasing antigenicity, immunogenicity and/or susceptibility to immune attacks [15]. For example, doxorubicin was demonstrated to promote so-called “immunogenic cell death” by inducing the translocation of the endoplasmatic reticulum protein calreticulin (CRT) to the surface of dying CT26 colon carcinoma cells, thus favouring phagocytosis by DCs [16]. Overall, the use of antineoplastic agents in combination with immunotherapies can be a rational approach to favour anti-tumoural responses.

Cancer antigenicity and susceptibility to immune attacks can be influenced by aberrant methylation patterns, which represent important elements in the regulation of cancer gene expression [17]. The impact of DNA methylation on cancer cells has been investigated by using hypomethylating agents that can induce important changes at the level of gene transcription and protein function. 5-Azacytidine (5-AZA) and Decitabine (DAC or 5’-aza-2’-deoxycytidine) are the best-known hypomethylating agents. DAC is a cytidine analog that is incorporated into the DNA and inhibits the enzyme DNA methyltransferase (DNMT) [18]. Concerning its immunosensitising effects, DAC was reported to increase MHCI expression and to restore the expression of certain tumour antigens by diverse cancers [19–25]. In particular, cancer testis antigens (CTAs) were affected; expression of these antigens is typically restricted to the testis and silenced in somatic cells. When CTAs are expressed by tumour cells they represent interesting tumour-associated antigens (TAAs) to be targeted by immunotherapies. Furthermore, enhancement of CTL-mediated tumour killing after DAC treatment was reported [21–24]. Indeed, in a murine mammary carcinoma model, this was directly linked to enhanced expression of the CTA P1A and lung metastases were significantly reduced after combined DAC treatment and adoptive transfer of specific CTLs [26]. In accordance with these findings, combinations of epigenetic drugs and immunotherapies such as immune checkpoint inhibitors have begun to be investigated in clinic trials for cancer treatment [27].

Regarding glioma antigenicity and hypomethylating agents, in human differentiated cell lines, DAC induced the expression of the highly immunogenic CTA NY-ESO-1 [28, 29]; moreover, the melanoma-associated antigen (MAGE) D4 CTA was shown to be up-regulated by DAC on human glioma cell lines [30]. Furthermore, DAC was reported to up-regulate molecules other than CTAs, including MHCI [29], important for visibility to T cells, and molecules relevant for sensitisation to immune effectors, such as cell death receptors including tumor necrosis factor-related apoptosis inducing ligand (TRAIL) receptors [28, 31]. Hence, DAC is a promising anti-neoplastic agent to be potentially used in combination with T cell-based immunotherapy in glioma. Nevertheless, these studies only used human GBM lines grown under conventional culture conditions that will represent GDCs rather than GICs (which require serum free culture in presence of neural stem cell growth factors). Therefore, whether DAC also induces phenotypic changes on GIC populations that will impact on their interaction with CTLs is an issue that requires elucidation.

In this *in vitro* study, we demonstrated that DAC modified the expression of key immune-related surface molecules on both GDCs and GICs. The expression of the cell death receptor FAS was significantly increased after DAC treatment and cell death mediated by a recombinant FASL compound was augmented, with a more prominent effect on GICs. Moreover, DAC sensitised glioma cells to CTL-mediated killing, regardless of their differentiation status.

## Materials and Methods

### Cell culture

Murine GL261-OVA cell line was kindly provided by Oliver Grauer (University of Munich, Neurology Department, Germany). GDC GL261-OVA were cultured in DMEM containing 4.5 g/l glucose and supplemented with 10% FCS, at 37°C, 5% CO_2_. GIC GL261-OVA were cultured in DMEM-F12 GlutaMAX™ supplemented with B-27 (Invitrogen), 30 Units/ml heparin (Bichsel), EGF 20 ng/ml (Life Technologies), bFGF 20 ng/ml (Life technologies) at 37°C, 5% CO_2_. During routine culture 200 μg/mL G418 (Geneticin, Life technologies) was added in order to maintain selection. GDC and GIC GL261 glioma cell lines were maintained under the same culture conditions described above for GL261-OVA cells. For cell culture, GDCs cells were detached from plastic with accutase (SIGMA); accutase was also used to dissociate GICs (incubation at 37°C, 5 minutes). Where indicated, cells were incubated with recombinant murine IFN-γ (Immunotools). All the cell lines were tested mycoplasma-negative.

For manipulation of cells incubated at 5% or at 1% of oxygen, a Ruskinn InVivo2 300 Workstation was used. All media and buffers utilized for the experiment were pre-equilibrated before use at the corresponding oxygen concentrations using hypoxic chambers (Billups-Rothenberg, Inc.; gas mixtures composed of 87% N_2_/8% CO_2_/5% O_2_, or 91% N_2_/8% CO_2_/1% O_2_). Cells were kept inside the hypoxic chambers at 37°C for incubation periods.

### Mouse survival studies

Intracranial glioma cell implantation was performed in syngenic C57BL/6J female mice using a stereotaxic apparatus (Stoelting, Indulab, Switzerland). Mice were anesthetized with a mixture of Ketamine 80 mg/kg (Warner-Lambert, Baar, Switzerland) and Rompun 10 mg/kg (Bayer, Leverkusen, Germany). Pre- and post-operative analgesia was provided by subcutaneous injection of Buprenorphine (0.05 mg/kg, 0.2 ml volume). In order to reduce cell leakage, the indicated number of cells was resuspended in methylcellulose (4 μl). The injection was performed using a Hamilton syringe. Cells were injected in the pallidum (2.6 mm lateral to the bregma and 3.5 mm below the skull). Mice were monitored daily and they were sacrificed by CO_2_ inhalation when symptoms were observed (≥15% weight loss, irregular breathing, hunched back, decreased activity/prostration, paresis/paralysis, convulsions).

### T cell proliferation and cytotoxicity

All mice used in this study were T cell receptor (TCR) transgenic C57BL/6J females. Pmel-1 and OT1 mice were purchased from Charles River Laboratories. T cells from Pmel-1 mice express TCR specific for gp100 epitope restricted by MHC class I (gp100_25-33_). T cells from OT1 mice express TCR specific for Ovalbumin (OVA) epitope restricted by MHC class I (OVA_257–264_). After mouse sacrifice, spleen and lymph nodes cells were isolated and re-suspended at a final concentration of 10^6^/ml, then pulsed with 10 nM of the specific peptide (OVA or hgp100, for OT1 and Pmel-1 respectively). After 2 days, 50 IU/ml of human recombinant IL-2 was added and cells were diluted 1 in 2. The same procedure was repeated at day 4. Six days after CTL generation, cells were used in a 4 hour (h) or 20h CTL killing assay or re-stimulated with Dynabeads^®^ Mouse T-Activator CD3/CD28, at a ratio 1:1. For re-stimulation experiments, tumour cells were irradiated (100 Gy) and CTLs were labelled with carboxyfluorescein succinimidyl ester (CFSE) at day 6 and the read-out was performed 3 days later. Cytokine content (IFN-γ) from the supernatant of CTLs co-cultured with GL261 tumor cells was determined using the BD OptEIA sets for IFN-γ (BD Biosciences), according to the manufacturer's instructions. For CTL culture, cells were kept at at 37°C, 8% CO_2_ in DMEM containing 4.5 g/l glucose and supplemented with 6% FCS, 10 μM Hepes (Life Technologies), 20 μM β-mercaptoethanol, 50 IU/ml of human recombinant IL-2 (Life Technologies). All animals used in this study were between 8 and 10 weeks of age. All experimental procedures used have been reviewed and approved by the institutional and cantonal veterinary authorities (Direction Générale de la Santé, République et Canton de Genève, authorisation: GE/146/14) in accordance with Swiss Federal law on animal protection.

### Antibodies and flow cytometry

The antibodies and the corresponding isotypes used in this study were all purchased from BD Pharmingen. For cell surface staining, we used the following mouse antibodies: anti-FAS (CD95) PE-CY7 (Jo2 clone), anti-MHCI (H-2K^b^) FITC (AF6-88.5 clone), anti-ICAM-1 (CD54) APC (3E2 clone), anti-PDL1 (CD274) PE (MIH5 clone). For cell staining, Fc Receptor blocking was always performed and dead cells were excluded (LIVE/DEAD fixable dead cell stain: Thermo Fisher) prior to analysis. The median fluorescence index ratio (MFIR) was calculated as ratio of antibody MFI/ isotype MFI. Data were collected on a Gallios flow cytometer (Beckman Coulter) using Kaluza for Gallios software (Beckman Coulter, version1.0) and processed using Kaluza analysis software (Beckman Coulter, version 1.3). CFSE staining of CTLs (OT1 or Pmel-1) or GL261 (GICs and GDCs) was performed in PBS. After a first wash, cells were incubated for 3 minutes at RT with 10 μM CFSE (10^7^ cells/ ml). Cells were then washed twice in their culture media, re-suspended and counted.

### CTL killing assay

For CTL killing assay, *in vitro* activated CTLs (OT1 and Pmel-1) were used at day 6 and target cells were previously treated for 48h with 10μM DAC (Sigma). Prior to the test, where indicated, CTLs were incubated for 2h with 1μM CMA (Sigma) in Dimethyl sulfoxide (DMSO), or DMSO alone (Sigma-Aldrich). Target cells were labelled with CFSE, and plated in a 96 U-bottom well plate with CTLs at an E:T ratio of 10:1 (10^5^:10^4^ cells/well, final volume of 100 μl). The read-out was performed after 4h or 20h. Where indicated, the blocking FAS:Fc (human, rec., Adipogen) or the correspondent control Fc:IgG1 control (human, rec., Adipogen) was added at a final concentration of 10μg/ml. Cells were then harvested adopting the same protocol used for cell culture, transferred to a 96 V-bottom well plates and stained with LIVE/DEAD yellow (Life Technologies) and CD8-PE-CY7 (Biolegend). The percentage specific lysis was calculated with the following formula: (percentage CTL-induced cell death—percentage spontaneous cell death) / (100—percentage spontaneous cell death).

### FASL-mediated cell death assay

After 48h pre-treatment with 10μM DAC (Sigma), GL261 GICs and GDCs were plated with increasing concentrations of mFASL (human, multimeric, rec., Adipogen) or its corresponding control Adiponectin (mouse, rec., Adipogen)—10^4^ cells in 100 μl culture medium, 96-well plate U-bottom. After 20h, cells were harvested as described above and stained with 7AAD (BD Pharmingen) as described in the manufacturer’s protocol. The percentage cell death was calculated as (percentage FASL-induced cell death—percentage spontaneous cell death) / (100 –percentage spontaneous cell death).

### Glioma proliferation studies

For proliferation studies, GL261 GICs and GDCs were labelled with CFSE (10 μM) and treated for 48h with the indicated concentrations of DAC. Cells were then harvested and analysed by flow cytometry.

### Pyrosequencing analysis

Dry pellet was harvested from cell culture and DNA/RNA was extracted using AllPrep DNA/RNA Mini Kit (Qiagen). After the bisulfite conversion of the DNA using the EpiTect Fast DNA Bisulfite Kit (Qiagen), a mouse LINE-1 PCR was performed using primers specific for 5 individual CpG site in the LINE-1 promoter region (EpigenDx) to analyse the methylation levels. The methylation status was measured by pyrosequencing technology on a PyroMarkQ24 sequencer with the PyroMark Q24 SW 2.0 software (Qiagen).

### Quantitative Real-Time PCR and Western Blot

RNA extraction from dry pellets collected from cell culture was performed using the RNeasy kit (Qiagen) according to the manufacturer’s instructions. Reverse transcription was done using PrimeScript reverse transcriptase (Takara Clontech). The assay was performed in 384-well plates using a pipetting robot Biomek 2000 (Beckman Coulter). The primers used are: Gfap (F: CTGGAGGTGGAGAGGGACAA, R: TGGTTTCATCTTGGAGCTTCTG), Met (F: AGAAGTTCACCACCAAGTCAGATG, R: CCCAGAGGAGCACACCAAAG), Olig2 (F: CAAATCTAATTCACATTCGGAAGGT, R: CTAAGCTCTCGAATGATCCTTCTTTT), Fabp7 (F: ATGGCAAGATGGTCGTGACTCT, R: CTTTTCATAACAGCGAACAGCAA). GFAP protein concentration by GL261 cells was determined by BCA protein assay kit from Pierce (ThermoFisher). Briefly, 20 μg of extracted protein after dry pellet lyses was loaded per lane and analysed by SDS-PAGE followed by western blotting. Immune detection was performed using rabbit anti-GFAP antibody (Dako) with an overnight incubation at 4°C, followed by peroxidase-conjugated goat anti-rabbit IgG (Sigma) and mouse anti-β-actin (Sigma) with a 1h incubation at room temperature). Enhanced chemiluminiscence detection system SuperSignal West Pico (ThermoScientific) was used for analysis.

### Decitabine

Decitabine (Sigma) was dissolved in sterile water at a stock concentration of 55 mM and stored at -20°C accordingly to manufacturer’s instructions. Thaw-freeze cycles were avoided.

### Statistical analysis

Statistical analysis was done using Prism 5.0 software (GraphPAD). P values < 0.05 were considered statistically significant. Paired student t-test, unpaired student t-test, and one-way anova test (Dunnet’s multiple comparison test) were performed as indicated.

## Results

### GL261 GICs are poorly differentiated and form rapidly growing tumours

In order to characterise the GL261 GDC and GIC cell lines, we investigated their neural stem-cell marker expression *in vitro* and their tumourigenic potential. GL261 GDCs grew adherently whereas GICs formed neurospheres that grew in suspension (S1A Fig). To determine the level of differentiation of GL261 GDCs and GICs, we investigated the gene expression of selected stemness, oligodendrocyte and astrocyte markers (S1B Fig). The radial glial *Fatty Acid Binding Protein 7* (Fabp7) was more expressed by GICs than GDCs, as previously reported [32, 33]. Furthermore, GICs expressed high levels of the *Oligodendrocyte transcription factor 2* (Olig2). In addition, the hepatocyte growth factor receptor Met, associated with glioma stem cell phenotype [34], was more expressed by GICs. To further confirm that GDCs and GICs had a diverse differentiation status, we tested the expression of the *glial fibrillary acidic protein* (Gfap), an astrocyte differentiation marker reported to be absent in GICs [33, 35, 36]. Accordingly, our results showed that Gfap was expressed at a higher level by GDCs, which was also validated at the protein level (S1C Fig). In addition to stem-like proprieties, another relevant characteristic of GICs is their enhanced rate of tumour growth *in vivo*. Since GL261 GICs induced terminal symptoms more rapidly than GDCs when injected orthotopically in syngenic C57BL/6 mice (S1D Fig), our results confirmed this key GIC characteristic. Similar results were obtained for OVA-transfected GL261 GDC and GIC cell lines used elsewhere in the study (data not shown). Overall, we showed that GL261 GDCs differ from GICs when considering cell morphology, gene and protein expression, as well as tumour initiating potential.

### Decitabine decreases DNA methylation and has direct cytostatic and cytotoxic effects on glioma cells

**Fig 1:**
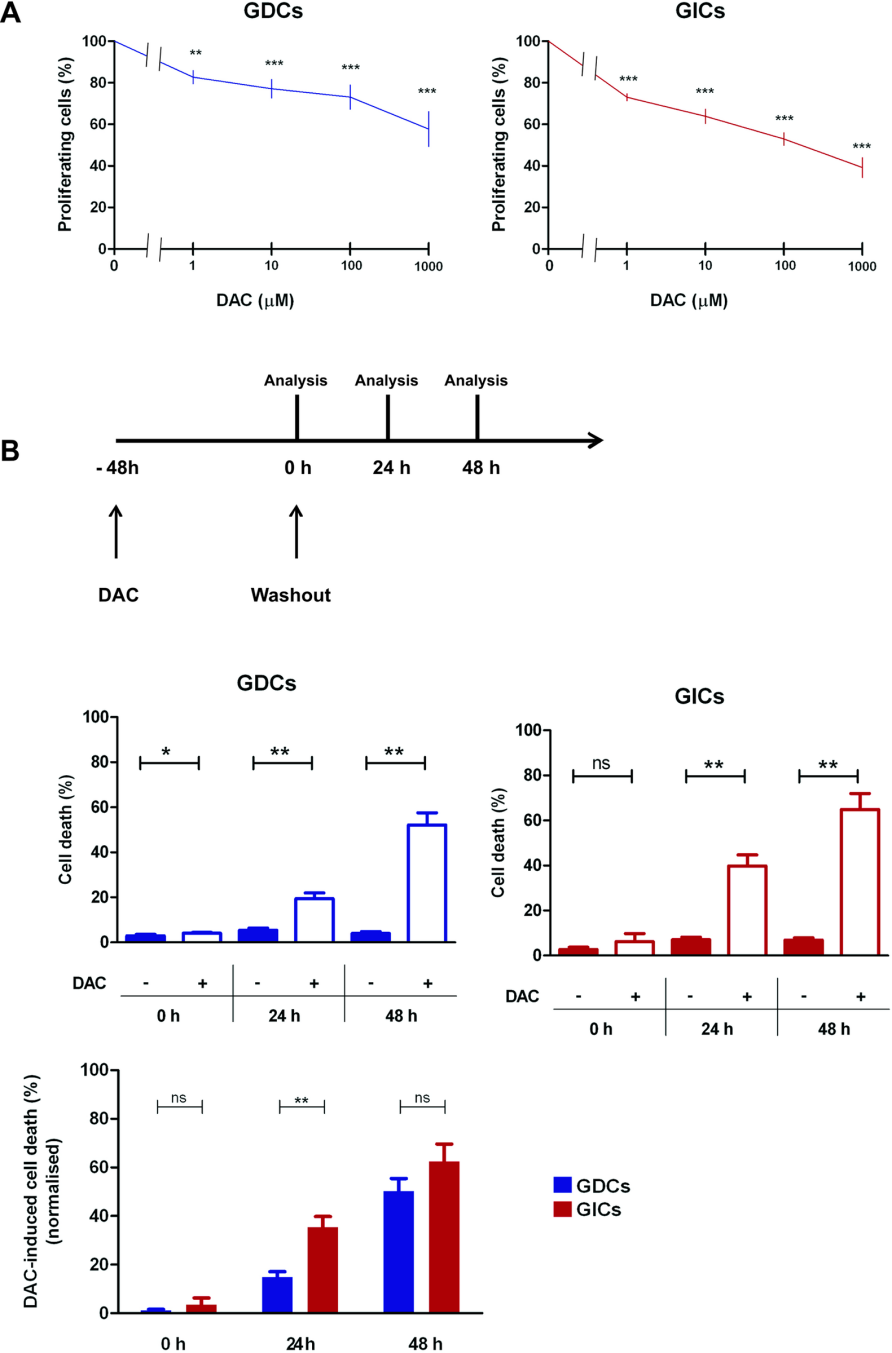
Decitabine has time-dependent cytotoxic effects and is cytostatic for GL261 glioma cells. (A) GL261-OVA GDC (GDCs) and GL261-OVA GIC (GICs) proliferation measured by CFSE dilution. Cells were CFSE-labelled, plated at the indicated DAC concentrations, and analysed 48h later. Proportions of proliferating GDC and GIC cells, normalised to vehicle controls are shown. *p<0.05;**p<0.01;***p<0.001. One-way Anova test (Dunnet’s multiple comparison test), n = 3. (B) GL261-OVA GDC (GDCs) and GL261-OVA GIC (GICs) percentage cell death measured by LIVE/DEAD fixable yellow dead cell stain after 48h DAC treatment (10 μM) at the indicated timepoints: cell death was measured at time 0 (0h), 24h and 48h after DAC washout (24h and 48h). In the bottom panel, DAC-induced cell death normalised to control is shown. Error bars represent SD. *p<0.05;**p<0.01;***p<0.001. Paired t-test performed (n = 3).

In order to assess the direct impact of DAC on both GL261 GDCs and GICs, we first validated DNA LINE-1 methylation decrease after treatment (S2 Fig); the effects were more pronounced on GDCs. We then investigated DAC cytostatic effects. We measured glioma cell proliferation by CFSE (carboxyfluorescein succinimidyl ester) dilution after 48h of DAC treatment at increasing doses (from 1 to 1000 μM) and we observed that DAC significantly impaired glioma cell proliferation of both GL261 GDCs and GICs in a dose-dependent manner (Fig 1A). Moreover, we measured the induction of cell death on both GL261 GDCs and GICs (Fig 1B). 48h after treatment, DAC had no impact on GL261 GICs and limited effects on GL261 GDCs. However, we did observe that DAC induced cell death on both GL261 GDCs and GICs a further 24h and 48h after DAC washout; GICs were more sensitive than GDCs at the 24h time-point.

### Decitabine augments GIC FAS expression and sensitises to FASL

**Fig 2:**
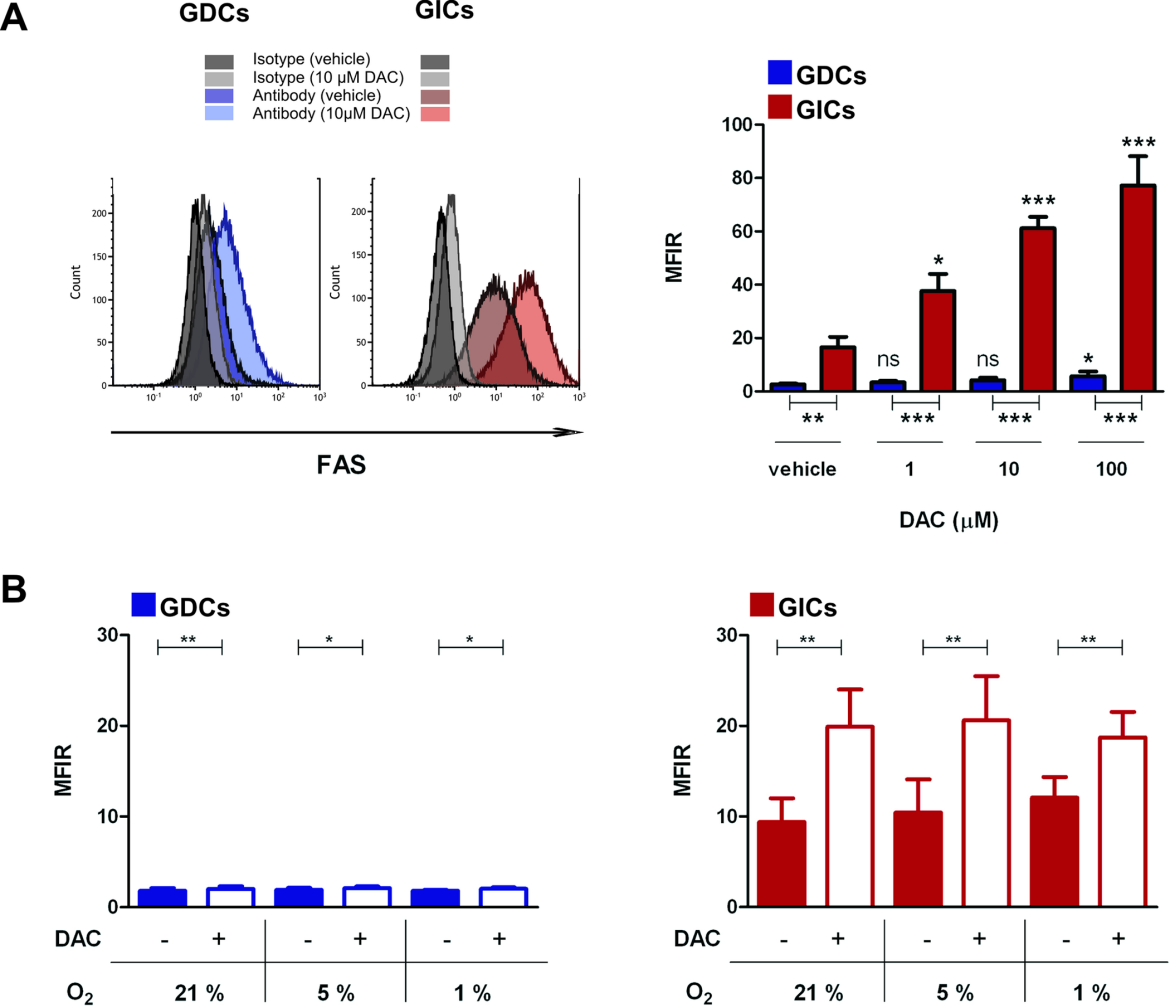
GL261 GIC FAS expression is high and increased by DAC. (A) Left panel: representative histogram of FAS staining of GL261-OVA GDC (GDC) and GL261-OVA GIC (GIC) (after prior 10μM DAC treatment of 48h), isotype control and anti-FAS antibody staining for each condition are shown. Live cells were gated. Right panel: FAS expression calculated as Mean Fluorescence Index ratio (MFIR) (MFIR Antibody/MFIR Isotype) by GDCs and GICs after 48h DAC treatment. Live cells were gated. Error bars represent MFIR + SD. One-way Anova test (Dunnet’s multiple comparison test) was performed to compare the expression of FAS by the same cell type at different DAC concentrations (ns, *, *** above bars); unpaired t-test was performed to compare the expression of FAS between the two cell types (**, *** under X axis). p<0.05;**p<0.01;***p<0.001, n = 3. (B) FAS expression by GL261-OVA GDCs (GDCs) (left) and GL261-OVA GICs (GICs) (right) calculated as MFIR. Cells were incubated at the indicated oxygen fractions (21%, 5%, 1% corresponding to atmospheric, physioxic, and hypoxic) in the presence or absence of DAC (1 μM). Live cells were gated. Error bars represent SD. *p<0.05;**p<0.01;***p<0.001, paired t-test, n = 3.

Anti-cancer drug sensitisation of tumour cells to immune-mediated attacks can occur by different mechanisms. Here, we investigated whether expression of the death receptor FAS was modulated by DAC on glioma cells. FAS expression by GL261 GDCs was only marginally up-regulated by DAC at the highest dose of DAC tested; in contrast, FAS expression was increased in a dose-dependent manner by GL261 GICs (Fig 2A). GL261 GICs expressed constitutively higher levels of cell-surface FAS than GDCs and this difference was maintained after DAC treatment. Moreover, we detected mRNA upregulation of other death receptors such as *Trail-R2*, as well as tumor necrosis factor receptors (*Tnfrsf1a* or *Tnfrsf1b*) on DAC treated GL261 GDC or GIC (data not shown).

In order to test if cell-surface FAS expression was also modulated by DAC under *in vivo* relevant oxygen fractions, we investigated FAS expression by GDCs and GIC at 1% O_2_ (i.e. hypoxia, condition of oxygen deprivation likely to occur at the brain tumour site) and at 5% O_2_ (i.e. physioxia, found for example in well oxygenated zones of tumour). We demonstrated that the increase in FAS expression induced by DAC was comparable at all of the different oxygen fractions tested, for both GL261 GDCs (Fig 2B, left panel) and for GL261 GICs (Fig 2B, right panel), which expressed higher levels of FAS than GDCs under all culture conditions.

**Fig 3:**
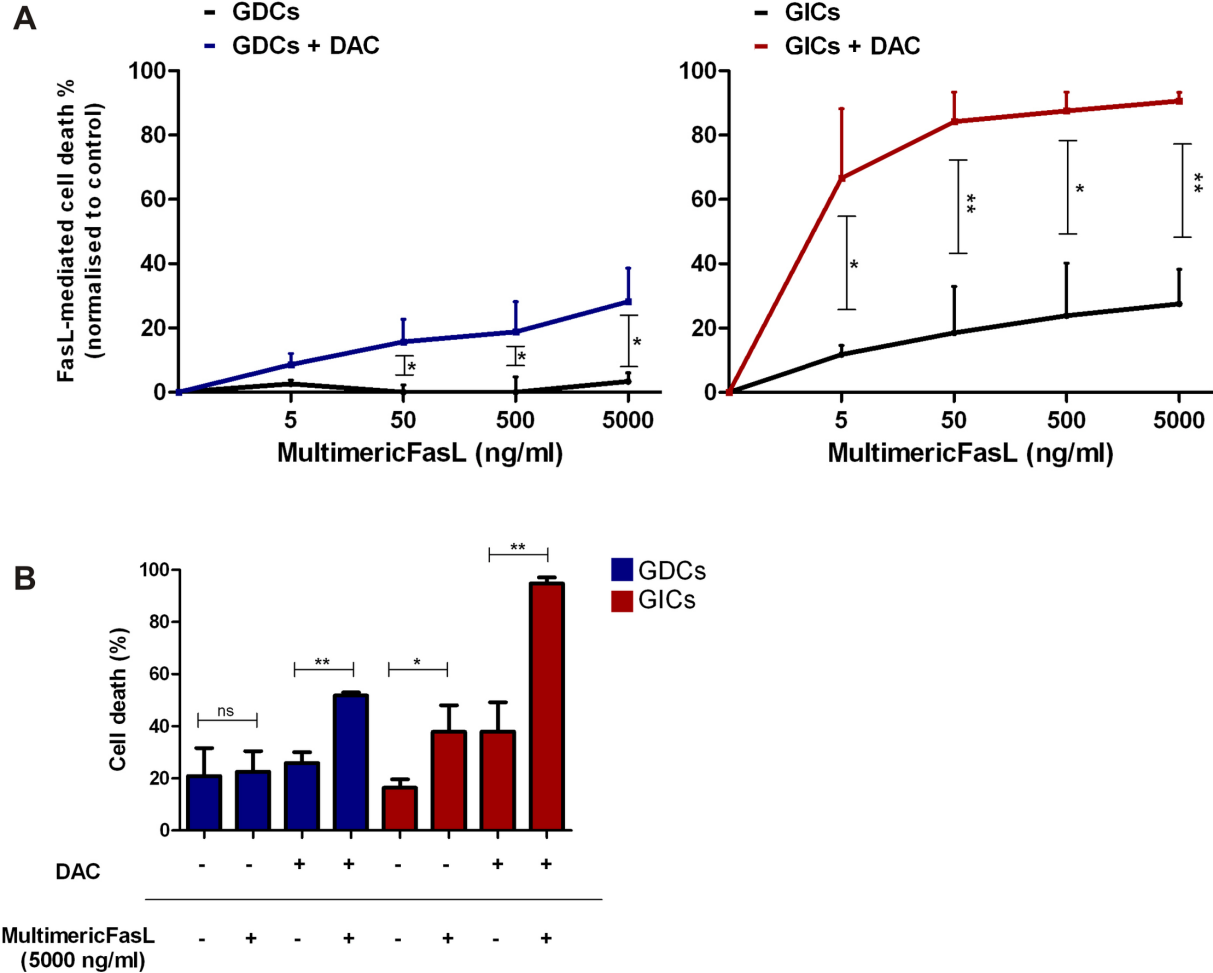
DAC enhances FASL-mediated cell death of GL261 glioma cells. (A) GL261-OVA GDC (GDC) and GL261-OVA GIC (GIC) FASL-mediated cell death normalised to untreated controls, measured by 7AAD staining. Cells were treated 48h with 10 μM DAC and then plated for 20h with increasing concentrations of the multimeric FASL or adiponectin (control). Error bars represent SD. *p<0.05;**p<0.01;***p<0.001, paired t-test, n = 3. (B) GL261-OVA GDC (GDC) and GL261-OVA GIC (GIC) FASL-mediated cell death without normalisation. The results for the highest concentration of multimeric FASL (5000 ng/ml) are shown. Error bars represent SD. *p<0.05;**p<0.01;***p<0.001, paired t-test, n = 3.

We then investigated whether the observed increase in surface FAS expression was sufficient to have functional consequences, i.e. whether it was associated with augmented FASL-mediated cell death. After DAC treatment, GL261 GDCs and GICs were incubated with increasing concentrations of a multimeric FASL (two trimeric FASL heads linked to an adiponectin tail), and cell death was assessed 20h later. DAC treatment augmented FASL-mediated cell death by GL261 GICs under all conditions tested, whereas for GDCs an increase was observed only from 50 ng/ml multimeric FASL (mFASL) (Fig 3A). However, in the absence of DAC pre-treatment, FASL killed GL261 GICs, whereas GDCs were insensitive (Fig 3B). Overall, DAC sensitised GL261 GDCs to FASL-mediated cell death, and augmented FASL-mediated cell death by GL261 GICs.

### Decitabine impacts on glioma phenotype and has consequences on CTL interactions

**Fig 4:**
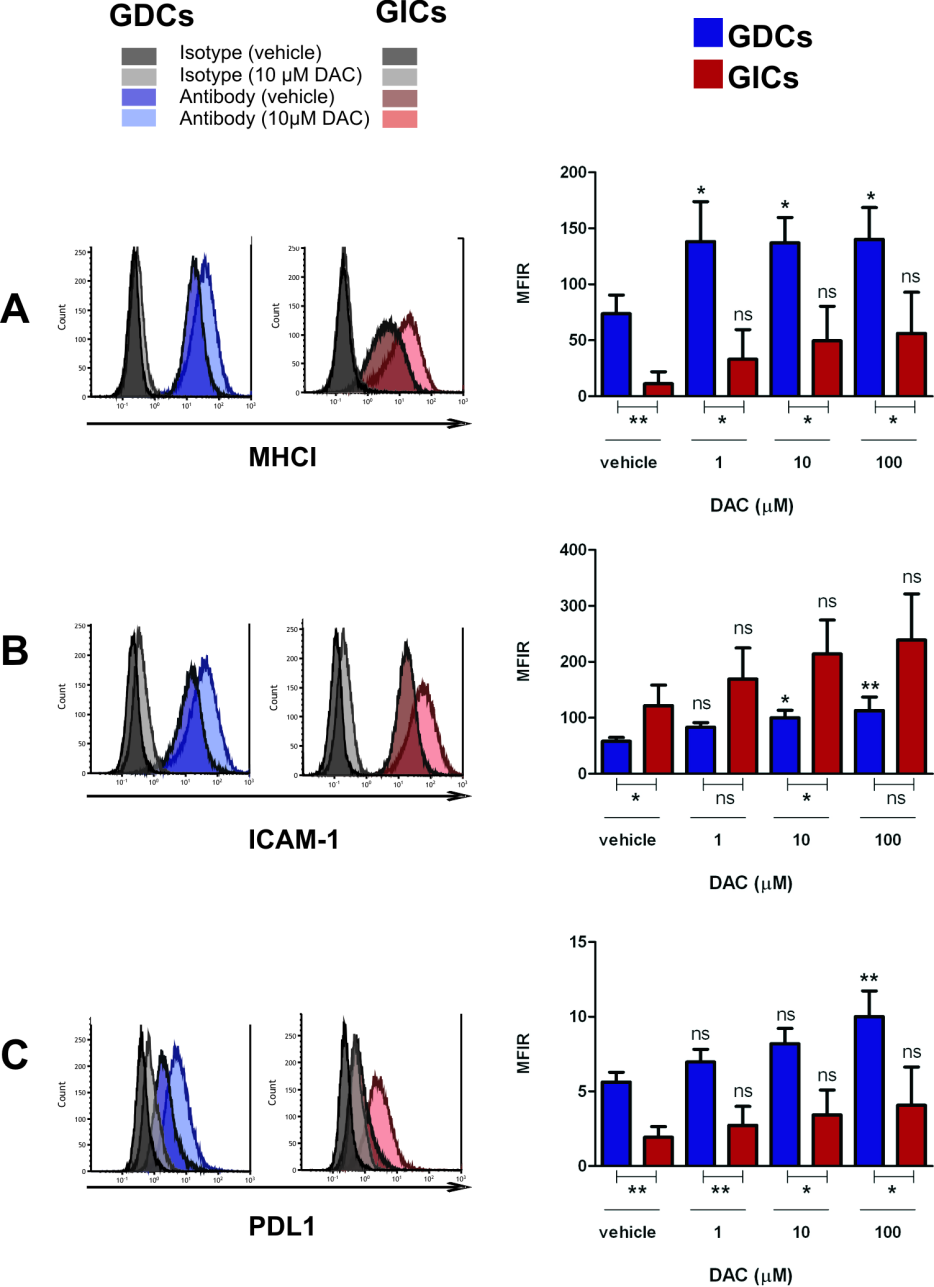
GL261 MHCI, ICAM-1 and PDL1 expression is impacted by decitabine. In the left panels, a representative histogram of GL261-OVA GDC (GDCs) and GL261-OVA GIC (GICs) expression of MHCI (H-2Kb) (A), ICAM-1 (B), and PDL1 (C) is shown (DAC used at 10μM, 48h incubation, both isotype control and antibody staining are shown). Live cells were gated. In the right panels, expression of each molecule (calculated as MFIR) by GL261-OVA GDCs (GDCs) and GL261-OVA GICs (GICs) after 48h DAC treatment is shown. Error bars represent SD. One-way Anova test (Dunnet’s multiple comparison test) was performed to compare the expression of MHCI, ICAM-1 or PDL1 by the same cell type at different DAC concentrations; unpaired t-test was performed to compare the expression of MHCI, ICAM-1 or PDL1 between the two cell types; p<0.05;**p<0.01;***p<0.001, n = 3.

DAC treatment of glioma cells was tested for its impact on the expression of selected surface molecules (i.e. MHCI and ICAM-1) considered important for CTL-target cell binding. In addition, the expression of PDL1, which could potentially have negative consequences on CTL functions, was also investigated. Taken as a whole, we observed that DAC had the tendency to increase the expression of all investigated molecules, by both GL261 GDCs and GICs (Fig 4). Furthermore, an increase in the expression of the same molecules was observed after Interferon-γ treatment (S3 Fig).

The constitutive expression of MHCI, ICAM-1 and PDL1 was different between GL261 GDCs and GICs: MHCI and PDL1 were more expressed by GL261 GDCs, whereas ICAM-1 expression was generally higher on GL261 GICs. After DAC treatment, these differential expression levels were maintained. In contrast, CD44 expression was not affected by DAC treatment, showing that DAC-induced phenotypic changes were not global (data not shown).

**Fig 5:**
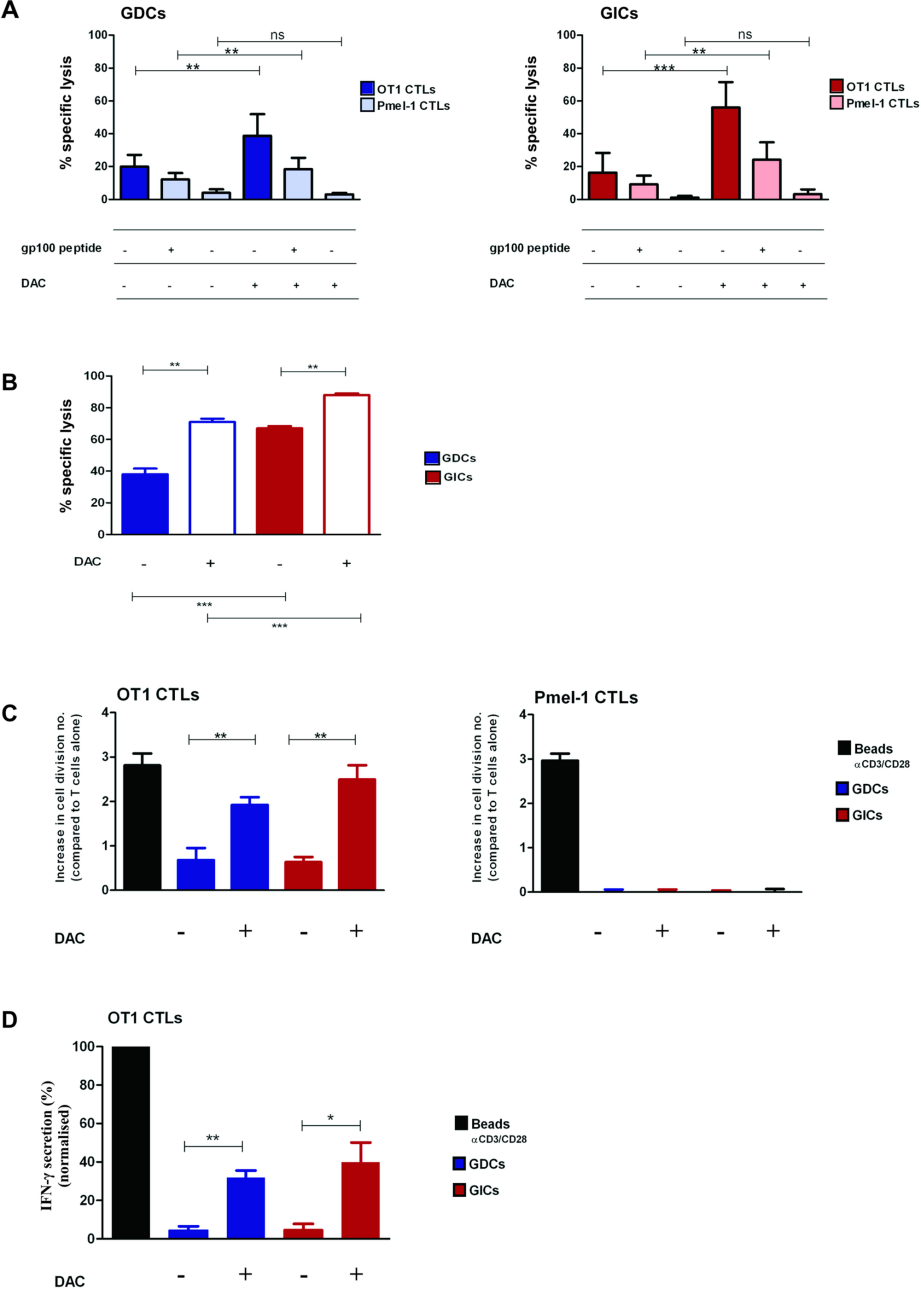
Decitabine treatment of GL261 glioma increases CTL-mediated killing and enhances CTL reactivation. (A) 4h killing induced by OT1 or Pmel-1 CTLs on GL261-OVA GDCs (GDCs) and GL261-OVA GICs (GICs). Target cells were plated for 48h with DAC (10μM) and then labeled with CFSE. When target cells were incubated with Pmel-1 CTLs, they were pulsed 1h with 1μg/ml gp100 peptide before the assay. The Effector:Target (E:T) ratio used was 10:1. Live cells were discriminated by LIVE/DEAD fixable yellow dead cell stain. Error bars represent SD. *p<0.05;**p<0.01;***p<0.001, paired t-test, n = 6. (B) 20h killing induced by Pmel-1 CTLs on GL261-OVA GDCs (GDCs) and GL261-OVA GICs (GICs). Target cells were pulsed 1h with 1μg/ml gp100 peptide before the assay. The protocol is described in (A). Error bars represent SD. *p<0.05;**p<0.01;***p<0.001, Paired t-test performed between untreated and treated groups; unpaired t-test performed between GICs and GDCs, n = 3 (C) OT1 and Pmel-1 CTL increase in cell division number after incubation with irradiated GL261-OVA GDCs (GDCs) or GL261-OVA GICS (GICs) calculated as difference in cell division number compared with CTLs incubated alone. CTLs were labeled with CFSE and co-cultured with irradiated GDCs or GICs that were previously treated with Decitabine (10μM, 48h). The number of T cell divisions was assessed by cytofluorometry (CFSE fluorescent peaks of gated events). CTLs were identified by CD8 staining. As positive control, anti-CD3/CD28-coated Dynabeads (Beads αCD3/CD28) were used. Error bars represent SD.*p<0.05;**p<0.01;***p<0.001, paired t-test, n = 3 (D) IFN-γ secretion by OT1 CTLs after incubation with GL261-OVA GDCs (GDCs) or GL261-OVA GICS (GICs). Values were normalised to positive controls (181 ng/ml +/- 84.9). Error bars represent SD.*p<0.05;**p<0.01;***p<0.001, paired t-test, n = 3.

With the aim of testing if the observed effects of DAC on GL261-OVA GDCs and GICs could impact on CTL-mediated killing, a cytotoxic assay was performed. We used either OT1 CTLs that recognise the OVA transgene constitutively expressed by GL261-OVA, or Pmel-1 CTLs that only specifically recognised GL261-OVA after pulsing with gp100 peptide. Under the conditions used for the test (with only modestly activated CTLs), there was low but significant killing of GDCs and GICs. We observed that after DAC pre-treatment, the CTL-mediated killing of both GL261-OVA GDCs and GICs was augmented (Fig 5A), with highest cytotoxicity achieved against GICs. Furthermore, this augmentation was observed when either OT1 CTLs (OVA-specific) were used, or Pmel-1 CTLs were used but with gp100-pulsed targets. Therefore, this enhancement was unrelated to particularities of the CTLs used but it could be related to modulation of elements involved in antigen processing and/or expression. At a later time point (20h), DAC augmented killing of gp100 peptide-pulsed GDC and GIC by Pmel-1 CTLs (Fig 5B). We also tested OT-1 CTLs at 20h, but with these cells, killing was extremely elevated under all conditions and so we could not discriminate any benefit of pretreatment (data not shown). Then, we investigated which mechanism was used by CTLs to kill the GL261-OVA target cells. In order to inhibit the CTL Perforin/Granzyme B pathway, we used the Granzyme inhibitor Concanamycin A (CMA) [37]. For all the tested conditions, we observed a partial inhibition of the CTL-mediated killing (S4A Fig). To then test whether any residual killing not blocked by CMA could be FASL mediated, we added a FAS:Fc fusion protein to the cells to block FAS/FASL binding (S4B Fig). No differences were observed, leading to the conclusion that FAS/FASL binding did not influence the observed CTL killing. We also tested whether either the Perforin/Granzyme B pathway or the FAS-FASL pathway could be blocked at 20h, but neither reagent had any impact on cytotoxicity, making conclusions difficult to draw for this time point (data not shown). Next, we tested if the observed changes on glioma surface phenotype also had an impact on T cell expansion and particularly on the capacity of glioma cells to re-activate CTLs (Fig 5C). GL261-OVA GICs and GDCs were treated with DAC (48h, 10 μM) and after irradiation they were co-cultured with CTLs from OT1 mice or Pmel-1 mice. 3 days later CTL proliferation and viability were measured. Both GL261-OVA GDCs and GICs were able to induce OT1 CTL re-activation, and pre-treatment with DAC increased this capacity (Fig 5C, left panel). The re-activation was antigen specific: Pmel-1 CTLs did not proliferate when in contact with GL261-OVA GDCs or GICs (in the absence of the gp100 peptide) (Fig 5C, right panel). We also measured cytokine release by CTLs after re-activation; DAC pretreatment of both GL261-OVA GDCs or GICs elicited enhanced secretion of interferon-γ by OT1 CTLs (Fig 5D).

Overall, we demonstrated that the hypomethylating agent DAC had an impact on both GDC and GIC cell surface phenotype, favouring cell death mediated by a recombinant FASL compound or by glioma-specific CTLs. Moreover, CTL proliferation was augmented. Consequently, the use of decitabine for glioma represents a promising immunosensitising strategy that can be potentially exploited in the context of glioma sensitisation to T cell immunotherapy.

## Discussion

The use of anti-neoplastic agents in combination with immunotherapies to sensitise tumours to immune attacks is receiving warranted attention in translational cancer research. Among the multitude of possible approaches, epigenetic drugs are particularly promising, with the expression of several immune-related genes noted to be influenced by methylation [24]. Several studies have analysed multiple glioma cell lines, which is important in view of glioma heterogeneity, but the focus to date has been on GDCs. Here, we investigated the responses of glioma cells to the hypomethylating agent DAC. We used the single murine glioma line GL261, but we used both GDCs and GICs in order to understand if DAC treatment affects the immunosensitivity of glioma cells with diverse differentiation levels and tumourigenic potential.

We firstly showed that DAC impaired cell proliferation of both GL261 GDCs and GICs (Fig 1A), suggesting that DAC cytostatic effect is regardless of cell proliferation rate–GICs being less proliferative (data not shown). Consistent with our findings, in a human lung carcinoma cell line, DAC was found to induce G2/M cell cycle arrest and increased the expression of p21 (a cyclin-dependent kinase inhibitor, cell cycle regulator) [38]. Furthermore, DAC cytostatic effects on the established U251 GBM cell line [39] and on one primary GBM cell line [31] were reported. Concerning the impact of DAC on cell viability, it is known that DAC effects on tumour cells are highly dose-dependent: for cultured leukaemia cells, it was reported that DAC-mediated re-expression of hypermethylated genes and changes in cell signalling pathways were achieved using DAC doses that did not induce acute cytotoxicity [40]. In our experiments, we principally analysed glioma cells after short exposure to DAC, in order to focus on consequences of methylation changes for immune interactions rather than toxicity.

It is known that expression of the cell death receptor FAS is heterogeneous in glioma, and glioma cell lines may differ in sensitivity to FASL-mediated cell death [41, 42] due to low surface FAS expression or due to the expression of apoptosis-regulating genes. In addition, FAS signaling can result in other functions, including the maintenance of stem cell-like programs in glioma cells [43]. Konkankit and colleagues showed that DAC induced a modest increase of FAS surface expression on the T98G human GBM cell line [28]. In our study, we showed DAC-induced FAS up-regulation by both GDCs and GICs which was also recapitulated under physiologically relevant low oxygen fractions, i.e. 5% and 1% O_2_. It is noteworthy that 21% O_2_ is the commonly used percentage of oxygen for cell culture, but it corresponds to the atmospheric environment rather than any physiologic conditions [44]. Moreover, we observed that GIC susceptibility to FASL-mediated cell death was higher than for GDCs (Fig 3A), and one can speculate that this result is in accordance with the higher level of FAS surface expression by GICs. Nevertheless, other effects of DAC at the level of the FAS signalling pathway or on the expression of other cell death receptors such as those for TRAIL cannot be excluded. Indeed, it was reported that DAC augmented caspase 8 expression and TRAIL receptor expression by primary cultures and established GBM cell lines [31]. Therefore, if extrapolating our results to an *in vivo* situation, targeting FAS surface receptor could be suggested as a therapeutic approach that would preferentially affect GICs. In accordance with our results, an immunotherapeutic approach that would merit investigation is the administration of a synthetic FASL after glioma sensitisation with DAC. A hexameric FASL (APO010) was tested by Eisele and colleagues in an orthotopic U87 murine model, and they observed that intratumoural delivery was necessary for improving symptom-free survival [41]. A Phase I dose finding clinical study of APO010 in patients with solid tumours (NCT00437736) has been completed recently, in which APO010 was administrated intravenously, although primary brain tumour patients were excluded.

In addition to cell death mediated by a recombinant FASL compound, we also demonstrated that DAC sensitised glioma cells to CTL-mediated killing. Importantly, the DAC-enhanced CTL killing remained strictly antigen-specific. This is a key result since off-target effects are important to avoid for T cell immunotherapies in a critical organ such as the brain. The observed DAC-mediated augmentation of ICAM-I and MHCI may have been responsible for increased CTL killing, through formation of a more functional cytotoxic immunological synapse (it is likely that binding of TCR/MHCI and LFA-1/ICAM-1 cooperates to stabilise the cytotoxic synapse). In our studies the principle cell death pathway we demonstrated to be used by the CTL clones was the Granzyme B pathway, and not the FAS-FASL pathway. However, one cannot exclude that *in vivo* other CTL populations would preferentially use the FAS/FASL pathway [45]. An additional phenotypic change we observed was an increased expression of the immunomodulatory molecule PDL1 by both GDCs and GICs; this did not overcome the positive interactions potentiated by DAC, despite the fact the CTL cells use in our tests expressed PD-1 (data not shown). Nevertheless, in the eventuality that PDL1 upregulation has greater consequences *in vivo*, use of the immune-checkpoint inhibitor anti-PD1/PDL1 in combination with decitabine might be envisaged.

We did not observe differences between CTL-mediated killing of untreated GL261 GDCs and GICs. This result is in contrast with our previous experiments using non-transfected GL261 GDCs and GICs [46] and with the findings by Avril and colleagues who reported that primary human glioblastoma cell lines cultured as GICs were more sensitive to killing by allogenic CTLs in comparison to differentiated cells [47]. Conversely, another study showed that human GDCs and GICs from GBM patients were comparable regarding their susceptibility to Perforin/Granzyme-mediated CTL killing [48]. Explanations for these disparate results could be because GIC phenotype characterization is not standardised (there are no universally accepted GIC markers), heterogeneity between different glioma cell lines, and besides, different experimental read-outs were used. We suggest that the modestly cytotoxic effector T cells we used in the short term (4h) cytotoxicity assay employed here were insufficient to discriminate differential sensitivity of GDCs or GICs, and under these suboptimal conditions (that might reflect the reality of many anti-tumour responses to natural tumor antigens), preferential GIC killing was only revealed by DAC pre-treatment. Indeed, under some conditions, we did observe preferential killing of GL261 GICs when the analysis was performed at a second time-point (at 20h, Fig 5B). This difference was observed independently of the DAC pre-treatment. Considering the fact that FAS was more highly expressed by GL261 GICs (both constitutively and after DAC treatment), we hypothesise that CTLs used the FAS/FASL pathway to induce glioma killing only at later time points, in accordance with the finding that FASL can be newly synthesised by CTLs after recognition of antigen presented by target cells [49].

In addition to increased CTL killing, we showed that DAC enhanced the capacity of glioma cells to induce proliferation and interferon-γ release of co-cultured, previously activated CD8 T cells (CTL re-activation) (Fig 5C and 5D). This finding is important if one considers that in an *in vivo* situation, only a limited number of CTLs reach the brain and may need to proliferate to provide significant anti-tumour activity [50]. Importantly, we demonstrated that both GDCs and GICs can induce CTL re-activation, and that DAC pre-treatment of glioma cells significantly favoured this process. This result is consistent with the change in key surface molecules we measured after DAC treatment, including MHCI. Furthermore, the observed CTL re-activation was antigen-dependent. This finding demonstrates that DAC would favour glioma killing and, simultaneously, induce positive signalling for CTL proliferation. Moreover, the process could be amplified by the enhanced interferon-γ release (Fig 5D), a cytokine able to further augment expression of the same surface molecules upregulated by DAC treated GL261 cells (S3 Fig).

Epigenetic modifications play an essential role in gene silencing, and the expression of as many as 160 genes was reported to be silenced by gene promoter methylation in human GBM cell lines [51]. Importantly, the promoter methylation status of the DNA repairing enzyme methylguanine-DNA methyltransferase (MGMT) is considered a predictive marker for the response of patients with GBM to TMZ therapy [52]. Consequently, when considering the use of DAC for gliomas, one has to take into account the impact of DAC on the MGMT methylation status. Moen and co-workers elegantly addressed this issue, using GBM lines with an MGMT unmethylated promoter: DAC sensitised these cells to TMZ. Nonetheless, TMZ sensitivity of GBM lines with a methylated MGMT promoter did not change after DAC treatment. In the case of an unmethylated MGMT promoter, DAC hypomethylated the cytosines belonging to the MGMT body gene, leading to MGMT decreased expression [53]. Thus, it is likely that a combination of promoter and gene body methylation influences MGMT protein expression. However, it would be prudent to restrict the use of DAC for GBM patients with an unmethylated MGMT promoter.

Another important aspect to be considered when using DAC for malignant gliomas is that this hypomethylating agent is known to cross the blood-brain barrier (BBB) [54]. However, concerning DAC delivery to the brain, there is still a lack of information in clinical applications due to the fact that up to now DAC usage has been mainly investigated for hematologic malignancies. Since the findings we present in this study are limited by the fact that they are uniquely *in vitro*, we did attempt to also perform *in vivo* experiments. However, in preliminary experiments using syngeneic immunocompetent mice, we did not find any treatment conditions that showed benefit of systemic administration of hypomethylating drugs in combination with tumour-specific CTL adoptive transfer. This correlated with no significant decrease in global methylation status in the brain tumour (data not shown). For these experiments we carefully established the maximum-tolerated doses, knowing that the main constraint associated with clinical use of DAC is its dose-limiting cytotoxicity due to myelosuppression [55, 56]. Nevertheless, Everson and colleagues recently showed a synergic effect of DAC in combination with adoptive transfer of NY-ESO-1-targeting CTLs [57]. However, in contrast to our syngeneic model, they used immunocompromised mice; the absence of immune cells was likely to be crucial for allowing the use of higher doses of DAC and, consequently, to obtain better glioma hypomethylation and sensitization to CTL attack. Another critical factor for drug penetration may be the level of blood brain barrier breakdown in particular glioma models. For future clinical development, low doses would be preferable and this would necessitate local delivery to the brain tumour site. In order to achieve brain tumour immunosensitisation, focused ultrasound disruption of the blood brain barrier could potentially be used as a future therapeutic approach to overcome problems related to drug delivery to the brain [58].

Our results suggest mechanisms by which glioma cells can be sensitized to immune attack. When these results are considered together with other published findings including *in vivo* studies, combining hypomethylating drugs with glioma immunotherapies such as adoptive cell therapy is an attractive approach to explore. Moreover, in addition to the changes in surface protein expression on glioma cells that we observed, evidence from other tumour types shows that epigenetic modulators can also derepress endogenous retroviral sequences and thereby increase immunogenicity through viral response mechanisms [59]. Thus, taken all together, these multiple consequences of DNA hypomethylation in tumour cells working through non-redundant mechanisms are highly complementary and may further enhance spontaneous or immunotherapy-induced anti-tumour immune responses in different cancer indications.

## Supporting Information

S1 Fig GL261 GICs grow as neurospheres, express stem-like markers and form rapidly growing tumours. (A) Morphology of cultured GL261 GDCs (left) and GL261 GICs (right) (magnification 35x, Zeiss Axiovert 100 microscope). (B) Quantitative Real Time PCR analysis of Fabp7, Olig2, Met, and Gfap normalized gene expression by GL261 GDCs and GICs. Error bars represent SD. *p<0.05;**p<0.01;***p<0.001, unpaired t-test, n = 3. (C) Western blot analysis of GFAP expression by GL261 GDCs and GICs. (D) Kaplan-Meier survival curve of C57BL/6J mice intracranially implanted with 1x10^5^ GL261 GDCs or 1x10^5^ GL261 GICs. Median survival: 30 days (GDCs) and 22 days (GICs). Log-rank (Mantel-Cox) test, *p<0.05;**p<0.01;***p<0.001.
(TIF)

S2 Fig DAC exposure affects LINE-1 methylation status by GL261 GDCs and GICs. LINE-1 DNA methylation status measured by pyrosequencing analysis on genomic DNA (after bisulfite conversion) of GL261-OVA GDCs (GDCs) and GL261-OVA GICs (GICs). 5 different CpG sites were analysed. Cell pellets were harvested 48h after treatment with DAC (10μM) or vehicle control. Error bars represent SD. *p<0.05;**p<0.01;***p<0.001, paired t-test, n = 3.
(TIF)

S3 Fig MHCI, ICAM-1, PDL1, and FAS expression by GL261 is upregulated by IFN-γ. GL261 GDCs and GICs were treated for 48h with 100 IU/ml of IFN-γ, or vehicle control and analysed by flow cytometry. The MFIR of surface expression for each marker is shown. Cells were live gated. Error bars represent SD. *p<0.05;**p<0.01;***p<0.001, paired t-test, n = 3.
(TIF)

S4 Fig Concanamycin A-treated CTLs show impaired killing of DAC-sensitised GL261 glioma cells. (A) OT1 CTL-mediated killing after 4h of GL261-OVA GDCs (GDCs) and GL261-OVA GICs (GICs). Target cells were plated for 48h with DAC (10μM), they were labelled with CFSE before the assay. Where indicated, CTLs were incubated 2h before the assay with 1μM of the V-ATPases inhibitor Concanamycin A (CMA) to block Perforin/Granzyme-mediated killing. The Effector:Target (E:T) ratio used was 10:1. Live cells were discriminated by LIVE/DEAD fixable yellow dead cell stain. Error bars represent SD. *p<0.05;**p<0.01;***p<0.001, paired t-test, n = 3. (B) 4h killing induced by OT1 CTLs on GL261-OVA GDCs (GDCs) and GL261-OVA GICs (GICs). The same experimental protocol described in (A) was followed. Where indicated, FAS:Fc fusion protein (10μg/ml) was added to the wells at the beginning of the assay. Error bars represent SD. *p<0.05;**p<0.01;***p<0.001, paired t-test, n = 3.
(TIF)
