# Supplementary figures and images for: Decitabine Treatment of Glioma-Initiating Cells Enhances Immune Recognition and Killing
Source: PLoS One. 2016 Aug 31;11(8):e0162105. doi: 10.1371/journal.pone.0162105 (PMC5007044; doi:10.1371/journal.pone.0162105)

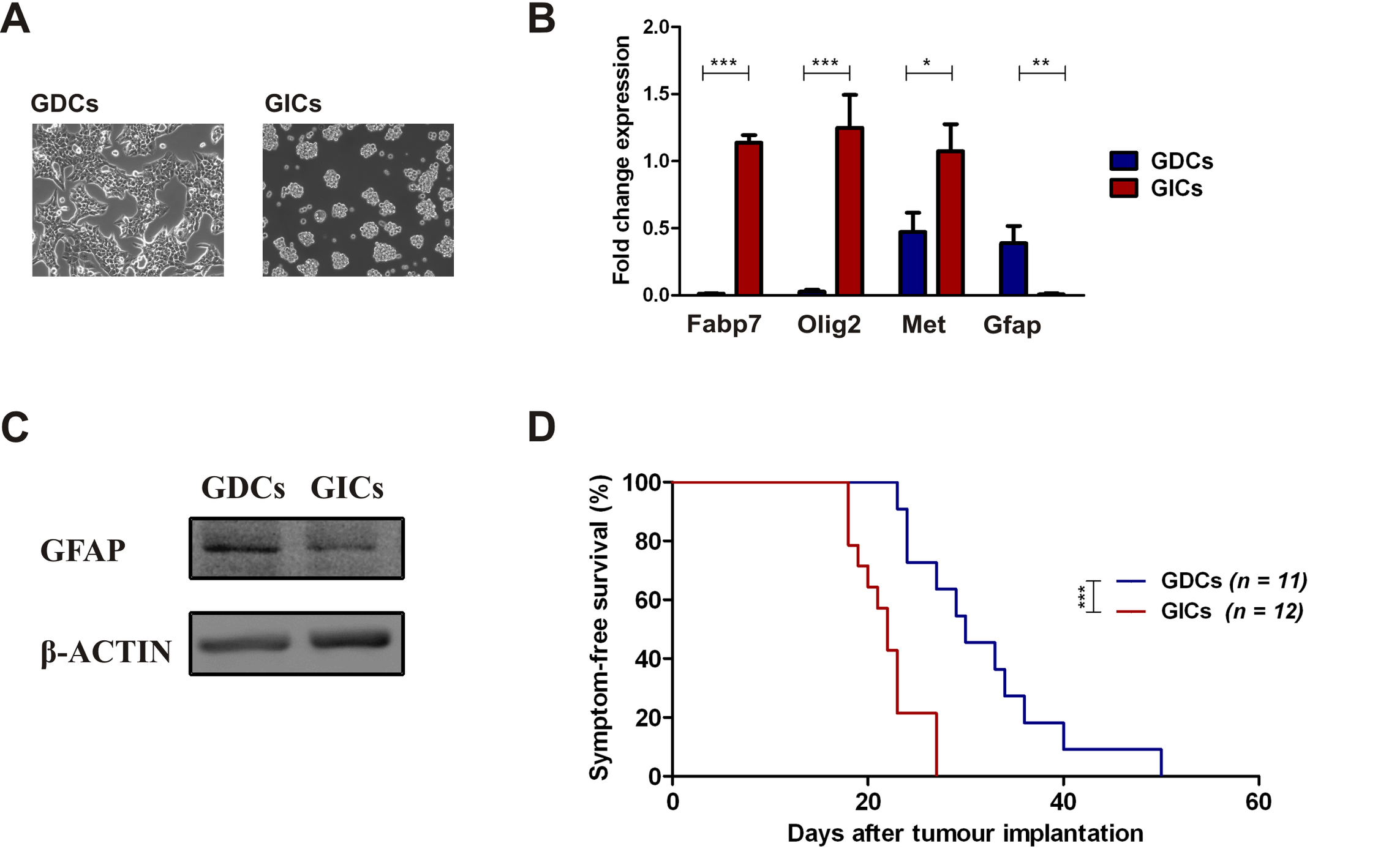

Supplement: S1 Fig — (A) Morphology of cultured GL261 GDCs (left) and GL261 GICs (right) (magnification 35x, Zeiss Axiovert 100 microscope). (B) Quantitative Real Time PCR analysis of Fabp7, Olig2, Met, and Gfap normalized gene expression by GL261 GDCs and GICs. Error bars represent SD. *p<0.05;**p<0.01;***p<0.001, unpaired t-test, n = 3. (C) Western blot analysis of GFAP expression by GL261 GDCs and GICs. (D) Kaplan-Meier survival curve of C57BL/6J mice intracranially implanted with 1x10^5^ GL261 GDCs or 1x10^5^ GL261 GICs. Median survival: 30 days (GDCs) and 22 days (GICs). Log-rank (Mantel-Cox) test, *p<0.05;**p<0.01;***p<0.001. (TIF) [file pone.0162105.s001.tif]

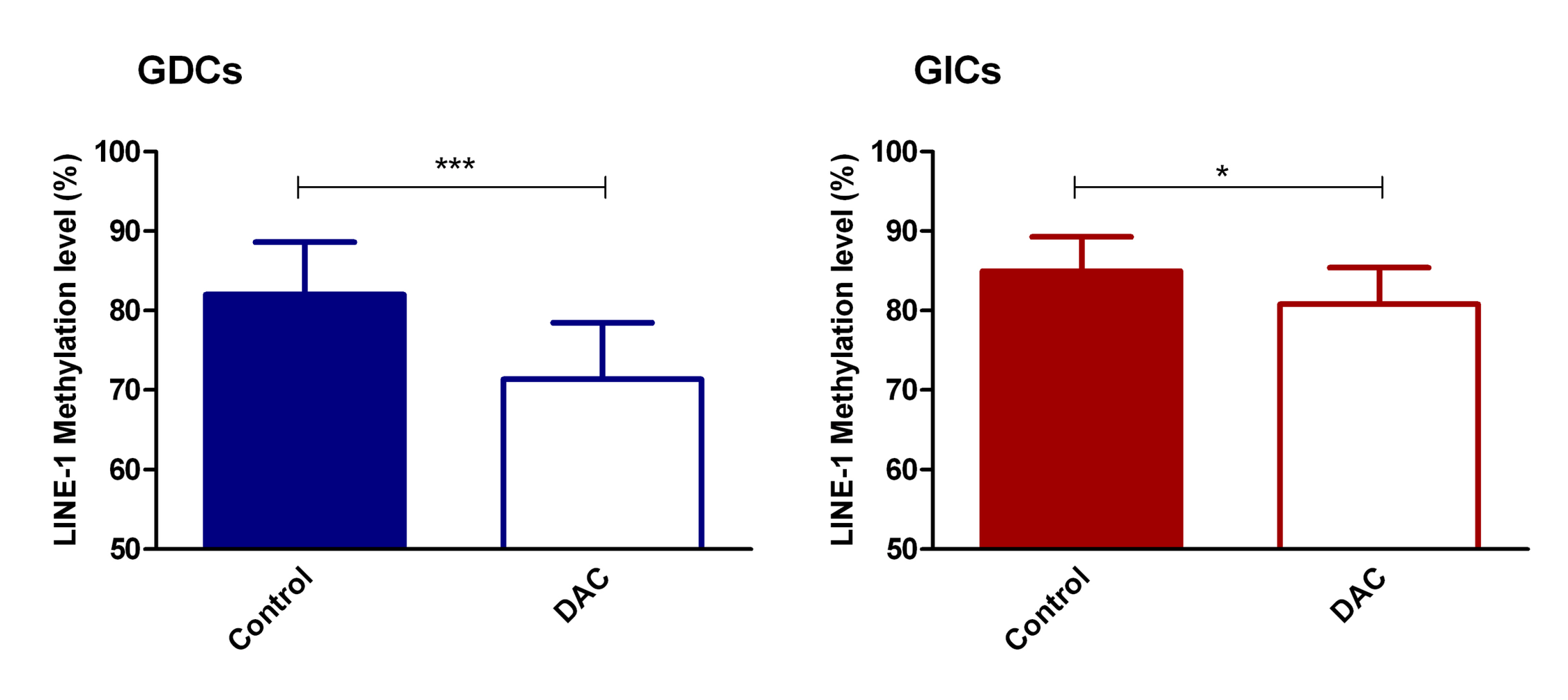

Supplement: S2 Fig — LINE-1 DNA methylation status measured by pyrosequencing analysis on genomic DNA (after bisulfite conversion) of GL261-OVA GDCs (GDCs) and GL261-OVA GICs (GICs). 5 different CpG sites were analysed. Cell pellets were harvested 48h after treatment with DAC (10μM) or vehicle control. Error bars represent SD. *p<0.05;**p<0.01;***p<0.001, paired t-test, n = 3. (TIF) [file pone.0162105.s002.tif]

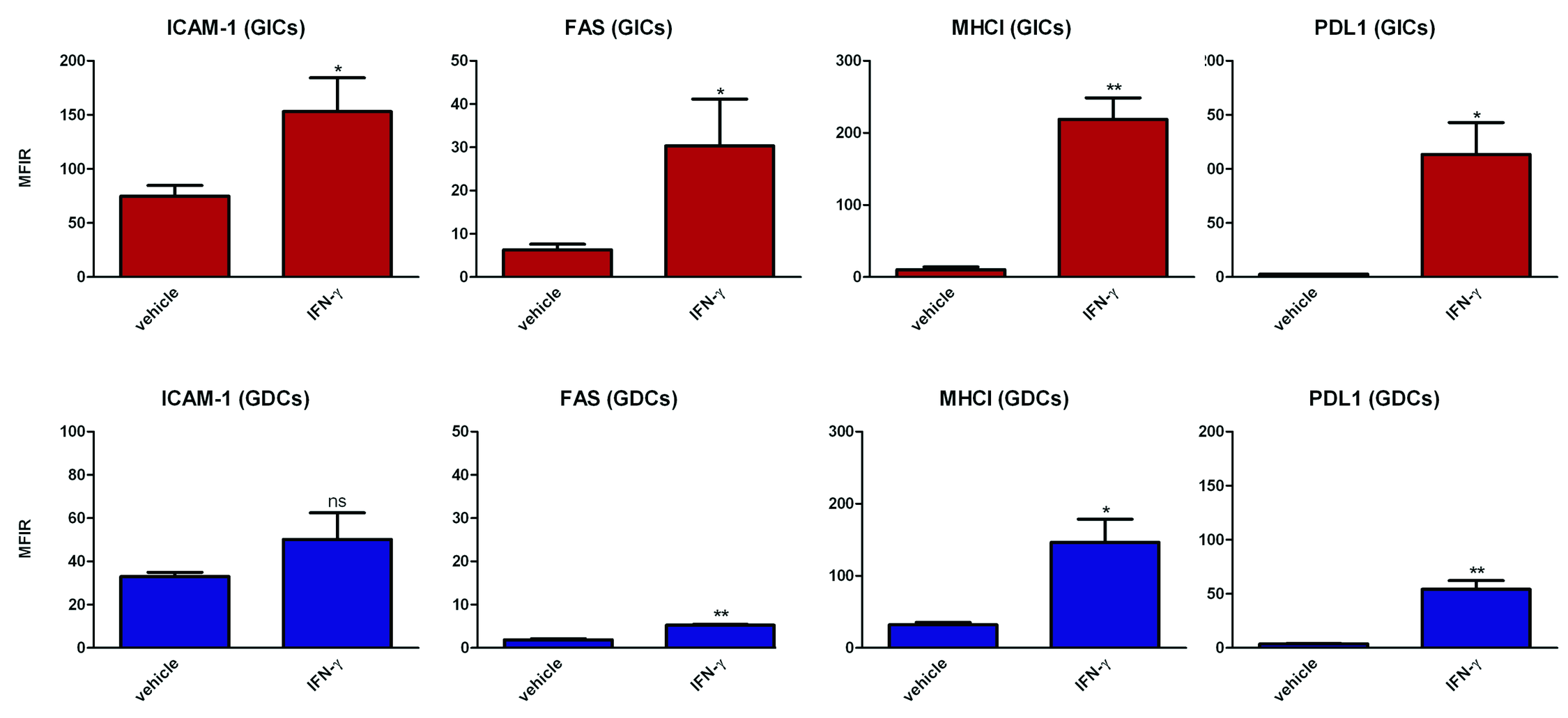

Supplement: S3 Fig — GL261 GDCs and GICs were treated for 48h with 100 IU/ml of IFN-γ, or vehicle control and analysed by flow cytometry. The MFIR of surface expression for each marker is shown. Cells were live gated. Error bars represent SD. *p<0.05;**p<0.01;***p<0.001, paired t-test, n = 3. (TIF) [file pone.0162105.s003.tif]

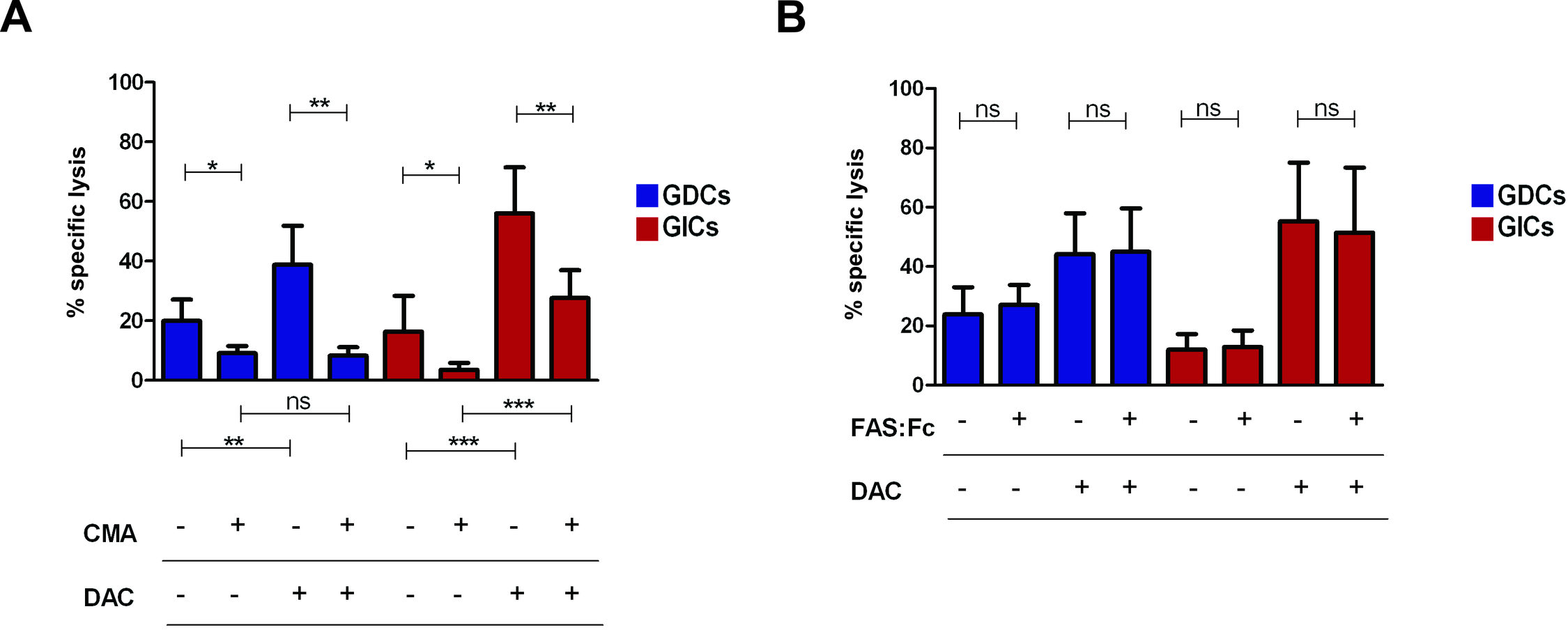

Supplement: S4 Fig — (A) OT1 CTL-mediated killing after 4h of GL261-OVA GDCs (GDCs) and GL261-OVA GICs (GICs). Target cells were plated for 48h with DAC (10μM), they were labelled with CFSE before the assay. Where indicated, CTLs were incubated 2h before the assay with 1μM of the V-ATPases inhibitor Concanamycin A (CMA) to block Perforin/Granzyme-mediated killing. The Effector:Target (E:T) ratio used was 10:1. Live cells were discriminated by LIVE/DEAD fixable yellow dead cell stain. Error bars represent SD. *p<0.05;**p<0.01;***p<0.001, paired t-test, n = 3. (B) 4h killing induced by OT1 CTLs on GL261-OVA GDCs (GDCs) and GL261-OVA GICs (GICs). The same experimental protocol described in (A) was followed. Where indicated, FAS:Fc fusion protein (10μg/ml) was added to the wells at the beginning of the assay. Error bars represent SD. *p<0.05;**p<0.01;***p<0.001, paired t-test, n = 3. (TIF) [file pone.0162105.s004.tif]
